# Critical Evaluation of Ayurvedic Plants for Stimulating Intrinsic Antioxidant Response

**DOI:** 10.3389/fnins.2012.00112

**Published:** 2012-07-26

**Authors:** Sunil Dutt Shukla, Maheep Bhatnagar, Sukant Khurana

**Affiliations:** ^1^Shri Bhogi Lal Pandya Government CollegeDungarpur, Rajasthan, India; ^2^Department of Zoology, University College of Science, Mohan Lal Sukhadia UniversityUdaipur, Rajasthan, India; ^3^Cold Spring Harbor LaboratoryCold Spring Harbor, NY, USA

**Keywords:** antioxidants, central nervous system, reactive oxygen species, traditional medicine, ayurveda, rasayana chikitsa, aging, herbs

## Abstract

Oxidative damage caused by free radicals plays an important role in the causation and progression of many diseases, including aging. Free-radical damage is countered by many mechanisms, including both active antioxidant enzymatic activity in our body and passive antioxidants. Antioxidant response of our body can accommodate increased oxidative damage in diseased states to a level but beyond that level, additional antioxidants are required to combat the increased stress. Apart from the regular dietary sources of antioxidants, many traditional herbal medicines demonstrate a potential to boost antioxidant activity. Rasayana chikitsa that deals with rejuvenation and revitalization is a branch of the Indian traditional medical system of ayurveda. We review some select herbs described in rasayana chikitsa that have been assessed by modern means for stimulating intrinsic antioxidant responses in humans. A critical evaluation of rasayana chikitsa will likely provide urgently needed, actual stimulants of our physiological antioxidant responses and not just more passive antioxidants to add to an already large catalog.

## Introduction

Oxidative stress arises when the antioxidant defense system of the human body is not entirely efficient. In response to a mild oxidative stress, our body can increase antioxidant defense but severe oxidative stress increases free radicals that can lead to cellular injury and death. Evidence suggests that free radicals, induced due to oxidative stress contribute to aging in general and various diseases, including neurodegenerative diseases, chronic inflammatory diseases, cancer, and cardiovascular disorders (Halliwell, 1994, 2001).

In this review, we will first provide a brief primer on oxidative damage and then focus on ayurvedic plants that have been assessed with contemporary approaches for their antioxidant potential, specifically wherever literature permits, to focus on the stimulation of bodily responses to counteract oxidative damage. The reason for us to focus specifically on stimulation of bodily antioxidant response is because of ambiguous evidence of health benefits of passive antioxidant supplements, while there being a dire need to counteract oxidative damage in many diseases. The purpose of this review is to attract attention of researchers to critically evaluate the potential of the traditional Indian herbal pharmacopeia.

## What is Oxidative Stress and How is it Counteracted?

Oxidative stress constitutes an alteration that is encountered when there is an imbalance between the production of reactive oxygen species (ROS) and the ability of the biological system to readily detoxify these reactive intermediates or easily repair the resulting damage. ROS can be a result of environmental insults, like cigaret smoking and ionizing irradiation but majority of ROS are produced in the normal functioning of the body, from intracellular sources (Sastre et al., 2000; Ames, 2004). Sources of one-electron reduction product of molecular oxygen include Fenton reaction, Haber–Weiss reaction, NAD(P)H oxidases (NOX), xanthine oxidase (XO), mieloperoxidase, cytochrome P450, mitochondria, and uncoupled nitric oxide synthase. Additionally peroxynitrite that yields secondary one-electron oxidants has been implicated in cellular damage (Radi et al., 2001).

Free radicals can interact with almost all the bio-molecules in different ways, altering their natural properties and making them more susceptible to damage. Such oxidative damage affects almost all components of the cellular machinery such as carbohydrates, lipids, proteins, and nucleic acids. Both the ROS and the end products of their reaction with various bio-molecules can cause DNA damage by altering its nitrogenous bases (Valko et al., 2004, 2006, 2007). Damage to the mitochondrial DNA, instead of nuclear DNA, is usually a more frequent cause of pathophysiology induced due to oxidative damage. ROS can also directly cause single and double strand breaks in the mitochondrial DNA strands, just as in nuclear DNA (Durham et al., 2006). Close proximity to the sites of ROS generation from the respiratory chain, lack of histone guard, and limited capacity for DNA-damage repair make the mitochondrial DNA 10–100 times more susceptible to oxidative damage as compared to the nuclear DNA (Ames et al., 1993; Floyd and Hensley, 2002).

Lipid peroxidation, is a free-radical mediated feed-forward chain reaction that apart from disrupting cellular membranes, can inactivate the other cellular components. Peroxidation involves the direct reaction of oxygen and lipid to form radical intermediates and semi-stable peroxides that in turn damage the cellular proteins, enzymes, nucleic acids, and membranes (Devasagayam et al., 2003). Malondialdehyde (MDA), which is a major end product of lipid peroxidation is frequently used as an index of the overall lipid peroxidation (Devasagayam et al., 2003). It cross-links proteins with nucleotides, which causes significant cellular damage (Sivalokanathan et al., 2006). The impact of oxidative damage on carbohydrates is relatively less studied. Free radicals such as react ^•^OH with carbohydrates by randomly abstracting a hydrogen atom from one of the carbon atoms, producing a carbon-centered radical. This leads to chain breaks in the carbohydrate molecules (Halliwell, 1994; Stohs, 1995; Griffiths and Lunec, 1996; Devasagayam et al., 2004). High abundance of proteins and their rapid rates of reaction with radicals and excited-state species, including singlet oxygen, make them the most susceptible biological targets for oxidative damage within the cell (Gracanin et al., 2009). Protein oxidation occurs as part of normal regulatory processes, as a defense mechanism against oxidative stress, or as deleterious processes when antioxidant defenses are overcome (Barelli et al., 2008). Although most oxidized proteins that are functionally inactive are rapidly removed, some can gradually accumulate with time and thereby contribute to the damage associated with aging as well as various diseases. Lipofuscin, an aggregate of peroxidized lipids and proteins, is known to accumulate in the lysosomes of aged cells (Stadtman, 1992) and brain cells of the patients with Alzheimer’s disease (Perry et al., 2002).

Different tissues have different sensitivity to oxidative damage, based on differences in ROS production, antioxidant molecules, enzymes, and the regenerative capacity of the tissue, for example, the neuronal tissue in general is more susceptible to oxidative damage. The high lipid content of the nervous tissue, in the form of myelin, with abundance of unsaturated fatty acids and long chain fatty acids in cell membranes, coupled with its high aerobic metabolic activity, make it particularly susceptible to oxidative damage (Floyd and Hensley, 2002).

Harman (1956) suggested that free radicals could be responsible for biological aging. Since then numerous studies have supported as well as contradicted this mitochondrial free-radical theory of aging (Alexeyev, 2009; Lapointe and Hekimi, 2010; Kitazoe et al., 2011). While the evidence for a simplistic idea of aging may not be conclusive, most diseases associated with the human aging process are known to have a strong oxidative stress component and this antioxidant defense generally reduces with age (Ames et al., 1993). The accumulation of net damage due to oxidative stress over a period of time is considered responsible for many age related disorders, Alzheimer’s disease, Parkinson’s disease, rheumatoid arthritis, cancers, and cardiovascular disorders, etc. (Gutteridge, 1993; Raha and Robinson, 2000; Halliwell, 2011). The flood of ROS generated in the neuronal and glial mitochondria during ischemic insults can be acutely devastating to the brain tissue (Aliev et al., 2002). Antioxidant stress is a major, if not primary mechanism linking obesity and metabolic disorders, especially insulin resistance or diabetes in animal models (Styskal et al., 2012) and alleviation of many symptoms is being reported in many studies by a regular consumption of antioxidants (Muellenbach et al., 2008, 2009; Curtis et al., 2010). Similar associations have been made between cardiac health and oxidative stress (Dai et al., 2012).

## What are Antioxidants?

Cellular and tissue defense against oxidative damage can be both enzymatic and non-enzymatic. Superoxide dismutase [Mn-SOD, Cu/Zn-SOD, extracellular (EC)-SOD], catalase (CAT), glutathione paroxidase, glutathione reductase, peroxiredoxins are key enzymatic defenses and glutathione, thioredoxin, ascorbate (vitamin C), alpha-tocopherol (vitamin E), and uric acid are key non-enzymatic defenses (Poljsak, 2011). In generous terms, antioxidants also include carotenoids, manganese, reduced selenium, and alphalipoate, etc. (Smith et al., 1999; Ryan et al., 2010; Halliwell, 2011). This defense system is vulnerable to various environmental or external factors, which include pollution, drugs, radiation, and stress. Different antioxidants act to diminish the oxidative damage *in vivo* and their mechanism of action are highly varied.

## Dietary Antioxidants and Their Impact on Health

A number of dietary antioxidants exist in herbal sources that are collectively known as phyto-antioxidants. Many plants consumed either as regular diet or as medicinal herbs reasons contain phyto-antioxidants. Various epidemiological studies have demonstrated the beneficial effects of high intake of fruits and vegetables in combating oxidative damage and other aging related problems (Eastwood, 1999). It is not obvious that whether this effect is achieved due to passive antioxidants or by boosting bodily antioxidant responses.

Many plants contain large amounts of polyphenols that can play an important role in adsorbing and neutralizing free radicals, quenching singlet and triplet oxygen, and decomposing peroxides. The majority of the antioxidant compounds belong to the family of flavonoids, lignans, and catechins. Due to the inefficiency of the endogenous defense system, in some physiopathological situations, increasing the amounts of dietary antioxidants is useful to diminish the cumulative effects of oxidative damage (Sun et al., 2002). In addition to the above compounds found in natural foods, vitamin C, vitamin E, carotene, and tocopherol, found in dietary sources are known to possess significant antioxidant potential (Cai et al., 2004; Tsao and Akhtar, 2005). While consumption of fruits and raw vegetables has been found to be very beneficial, passive antioxidant therapies have largely yielded mixed results, with some studies also showing significant negative impact on human health (Bjelakovic et al., 2004). This negative effect could be due to the global down-regulation of ROS pathways that are required for our immune system, many intracellular signaling pathways and the maintenance of hormesis effect that might be crucial in maintaining the intrinsic antioxidant response of our bodies in balance (Poljsak, 2011). Additional problems can include poor bioavailability of many passive antioxidants (Siow and Mann, 2010; Patel et al., 2011; Wallace, 2011). Given these problems with passive dietary antioxidants two possible approaches are likely going to be fruitful: regulation of ROS production and selective stimulation of specific intrinsic antioxidant pathways. In recent years NADPH oxidases have been targeted for their therapeutic potential in regulation of ROS production (Drummond et al., 2011). In this review we will focus on the stimulation of bodily enzymatic antioxidant pathways but it is also possible that the plant sources that we discuss may in future be found to even help regulate the production of ROS and not just have antioxidant responses.

## Ayurvedic Plants with Potential of Providing Novel Antioxidant Stimulants

In the following text, we mention plants used in Indian pharmacopeia with their scientific name but we also provide English and Hindi names in parenthesis. Names in parenthesis, before the semi-colon are the English names and ones after, are the Hindi ones. Apart from the strictly dietary sources, many medicinal plants also provide antioxidants. Some such plants fall in both dietary and medicinal groups as they are used for both purposes. Few examples of plants used for both dietary and medicinal use that are rich in antioxidant effects are as follows: *Allium cepa* (onion; pyaaz), *A. sativum* (garlic; lahsuna), *Aloe vera* (Indian aloe, true aloe, medicinal aloe, Chinese aloe; ghritkumari), *Camellia sinensis* (green tea; chai), *Cinnamomum verum* (cinnamon; dal chini), *C. tamala* (Malabar leaf; tejpat), *Curcuma longa* (turmeric; haridra, haldar, haldi), *Emblica officinalis* (Indian gooseberry; amlaki), *Glycyrrhiza glabra* (liquorice; yashtimadhu, mulethi), *Ocimum sanctum* (holy basil; tulsi), *Terminalia bellerica* (beleric, bastard myrobalank; behda), *Tinospora cordifolia* (heart-leaved moonseed; guduchi), *Trigonella foenum-graecum* (fenugreek; methi), and *Zingiber officinalis* (ginger; adarak).

Apart from these dietary plants with medicinal value, ayurveda details strict medicinal plants that show strong antioxidant activities. “Rasayan Chikitsa” (Rasa = vital + Ayana = nourishment; chikitsa = treatment) is an important branch of ayurveda that deals with revitalization and rejuvenation in a holistic manner to slow aging and also to cure certain diseases. Sharangdhar in sixteenth century AD described Rasayanas as “Jaravyadhi Vinasanam” which literal means checking the advancement of age (Jara = age) as well as destroyer (Vinasanam = destruction) of disease (Vyadhi = disease; Rastogi, 2010). The modern scientific evaluation of some of the major Vayahsthapan (anti-aging) rasayanas and Jeevaniya (life-promoting) rasayanas shows that most of these anti-aging drugs have antioxidant properties, besides several other pharmacological actions (Rastogi et al., 1998; Anekonda and Reddy, 2005). Many passive antioxidants are already extracted and commercialized from dietary sources in addition to the synthetic compounds (Shahidi, 1997), so we have not cataloged over hundreds of plants that are generally rich in phyto-antioxidants. The examples we discuss are either known to have or have a good chance to have compounds that stimulate intrinsic antioxidant machinery of the human body. These plants also happen to have passive antioxidant compounds in large quantities that we mention in chemical constituents. The ultimate goal of this review is to emphasize the need of curing oxidative damage related problems by stimulating the intrinsic antioxidant machinery of the body, instead of just treating the symptoms using passive dietary antioxidants. Following are some select Auyrvedic plants with description of their sources, traditional uses, modern verification of some of their uses, and their antioxidant potential. A summary of these plants is presented in Table 1.

**Table 1:** **Plants with known or likely antioxidant stimulating effects**.

Plant	Antioxidant responses	Active antioxidant effects and known or putative compounds
*Withania somnifera*	Active stimulation and passive antioxidants	Stimulation due to sitoindosides VII–X and withaferin A (glycowithanolides; Bhattacharya et al., 1997; Vimal et al., 2010)
*Centella asiatica*	Active stimulation and passive antioxidants	Enhances glutathione levels, thiols, and antioxidant defenses (Shinomol and Muralidhara, 2008; Shinomol et al., 2010). Antioxidant in three pathways: superoxide free-radical activity inhibition of linoleic acid peroxidation and 2,2-diphenyl-1-picryl-hydrazyl (DPPH) radical scavenging activity (Vimala et al., 2003; Pittella et al., 2009). Reduces monamine levels that can spontaneously auto-oxidize to free radicals (Bindoli et al., 1992). Exact compounds remain to be identified
*Asparagus racemosus*	Active stimulation and passive antioxidants	Prevents decline in GPX activity and reduction in glutathione (GSH) content and reduces membrane lipid peroxidation and protein carbonyl content (Parihar and Hemnani, 2004). Compensates reduction in the superoxide dismutase, catalase, ascorbic acid, and lactate dehydrogenase levels and lowers the heightened MDA levels (Vimal et al., 2010). Exact compounds remain to be identified
*Acorus calamus*	Active stimulation and passive antioxidants	Decrease GSH and GST and increase dopamine receptors (Shukla et al., 2002). Increases the activities of major enzymes of the antioxidant defense system, especially SOD, CAT, and GPX and the levels of GSH and decrease in the formation of MDA (Sandeep and Nair, 2010). Exact compounds remain to be identified
*Bacopa monnieri*	Active stimulation and passive antioxidants	*Bacopa monnieri* extracts modulate the expression of certain enzymes involved in the generation and the scavenging of reactive oxygen species in the brain (Govindarajan et al., 2005). Exact compounds remain to be identified
*Celastrus peniculatus*	Active stimulation and passive antioxidants	Causes significant decrease in the brain levels of MDA and increases in levels of glutathione and catalase (Kumar and Gupta, 2002). Superoxide dismutase activity is unaffected by extracts but catalase activity is increased and MDA levels are reduced (Godkar et al., 2006). Exact compounds remain to be identified
*Convulvulus pleuricaulis*	Known passive antioxidants only	Given the antioxidant effects it is likely that there is likely stimulation of active antioxidant responses too that yet remains to be characterized
*Curcuma longa*	Known passive antioxidants only	Given the antioxidant effects it is likely that there is likely stimulation of active antioxidant responses too that yet remains to be characterized

### *Withania somnifera* (dunal; aswagandha, asgandhi, asoda, and amukkira)

#### Geographical distribution and ethnomedical description

*Withania somnifera* shrub that grows wild in India, is widely distributed across Africa, Europe, and Asia. It is commonly referred to as Ashwagandha (Ashwa = horse + Gandha = smell) in Sanskrit and Hindi because of its smell. In ayurveda, *W. somnifera* herbal remedy consists of the use of dried roots and stem bases of the plant. In traditional system it is used as anti-inflammatory and antitumor agent, immunity booster, and aphrodisiac for both men and women, though many of these effects have yet to be evaluated by modern means.

#### Chemical constituents

The chemistry of *W. somnifera* has been extensively studied and over 35 chemical constituents have been identified, extracted, and isolated (Rastogi et al., 1998). The biologically active chemical constituents are alkaloids (isopelletierine, anaferine), steroidal lactones (withanolides, withaferins), saponins containing an additional acyl group (sitoindoside VII and VIII), and withanolides with a glucose at carbon 27 (sitoindoside IX and X; Rastogi et al., 1998).

#### Active antioxidant and other roles

Animal stress studies have investigated the use of this herb as an antistress agent (Dadkar et al., 1987; Dhuley, 1998; Archana and Namasivayam, 1999), therapeutic in depression (Ramanathan et al., 2003) and explored its antioxidant properties (Panda and Kar, 1997). The active principles of *W. somnifera* – sitoindosides VII–X and withaferin A (glycowithanolides), have been tested for their antioxidant stimulant activity by measuring the levels of major free-radical scavenging enzymes, such as SOD, CAT, and glutathione peroxidase (GPX) levels in the rat brain frontal cortex and striatum (Vimal et al., 2010). Active glycowithanolides of *W. somnifera* show a dose dependent increase in activity of all these enzymes, to a level similar to seen with intraperitoneal administration of deprenyl, a known antioxidant (Bhattacharya et al., 1997). While, such strong active antioxidant effects have been reported, the exact active ingredients responsible for this stimulation of the innate antioxidant machinery remain to be identified (Bhattacharya et al., 1997). While large doses of ashwagandha may possess abortifacient properties and it can act as a mild central nervous system (CNS) depressant, in most cases ashwagandha appears to be very safe, with an LD_50_ of a 50% alcohol extract determined to be 1 g/kg in rats (Aphale et al., 1998; Williamson, 2002). Caution should be used with patients on anticonvulsants, barbiturates, and benzodiazepines due to its GABA-nergic and sedative properties (Mehta et al., 1991; Kulkarni et al., 2008; Bhattarai et al., 2010).

### *Centella asiatica* (Linn.) urban (Indian pennywort; brahmi, manduka parani, mandooki, divya, bhekaparni)

#### Geographical distribution and ethnomedical description

*Centella asiatica* is a small creeping herbaceous plant found in damp, shady places throughout the tropical regions of the world. *C. asiatica* finds mention in *Shsruta samhita* and is an important component of the Indian pharmacopeia (Nadkarni, 1910). In ayurveda, *C. asiatica* parts are considered useful in the diseases of skin, nervous system, and blood. The leaves are the only recognized part in traditional pharmacopeia of India, but many modern investigators have advocated the use of entire plant, root, twigs, leaves, and seeds in medicine (Hamid et al., 2002; Sudarshan, 2005).

#### Chemical constituents

*Centella* contains high concentration of metal ions potassium, calcium, phosphorus, iron, and sodium. It is low in protein, carbohydrate, fat, and crude fibers but rich in vitamin C, B1, B2, niacin, carotene, and vitamin A (Tee et al., 1997). The biologically active components of *Centella* plant are believed to be triterpenes and saponins (Loiseau and Mercier, 2000). The triterpenes of *Centella* are composed of many compounds including asiatic acid, madecassic acid, asiaticosside, madecassoside, brahmoside, brahmic acid, brahminoside, thankiniside, isothankunisode, centelloside, madasiatic acid, centic acid, and cenellic acid (Zheng and Qin, 2007). Among these triterpenes, the most important biologically active compounds are the asiatic acid and madecassic acid (Inamdar et al., 1996). *C. asiatica* contains triterpene glycosides such as centellasaponin, asiaticoside, madecassoside, and sceffoleoside (Matsuda et al., 2001). The content of *Centella*’s triterpene components can be affected by the location and diverse environmental conditions (James and Dubery, 2009). *Centella* also contains high total phenolic contents in the form of flavonoids such as quercetin, kaempherol, catechin, rutin, apigenin, and naringin and volatile oils such as caryophyllene, farnesol, and elemene (Qin et al., 1998).

#### Active antioxidant and other roles

Preliminary modern studies have demonstrated some beneficial effects in neuronal function, ulcers, liver functions, and immune responses (Warrier et al., 1996; Dash and Jounious, 1997; Frawley and Lad, 2004). *Centella* impedes brain aging and improves attention problems (Singh et al., 2008). Besides this, *Centella* is also reported to be useful in improving learning scores in young mice (Rao et al., 2005), providing protection against neurodegeneration (Ramanathan et al., 2007), neuroprotection against oxidative stress in the brain of prepubertal mice, enhancing glutathione levels, thiols, and antioxidant defenses (Shinomol and Muralidhara, 2008; Shinomol et al., 2010). *C. asiatica* extracts can influence the morphology of hippocampal CA3 and amygdalar neuronal dendritic arborization in neonatal rats (Rao et al., 2006, 2009) and can have anxiolytic effect in animals comparable to diazepam (Chen et al., 2006). Patients of anxiety necrosis showed reduced anxiety levels and showed improvement in the mental fatigue rate and immediate memory after a *C. asiatica* regimen (Bradwejn et al., 2000; Jana et al., 2010) and *Centella* attenuates d-galactose-induced behavioral, biochemical, and mitochondrial dysfunction by involving antioxidant and mitochondrial pathways in the senescent mice (Kumar et al., 2011). Brahminoside exhibits a CNS-depressant effect in mice and rats (Ramaswamy et al., 1970). In an old clinical study, *C. asiatica* regimen showed improvement in mental health of mentally challenged children (Rao and Rao, 1973). Whether these effects are predominantly due to active antioxidant stimulation, passive antioxidants, or other mechanisms is not fully known. *C. asiatica* extracts have been reported to increase brain GABA levels (Chatterjee et al., 1992), suggesting neuromodulation to also underlie some of its neuroactive actions. Beside the triterpenes, the antioxidant protection effect of *Centella* is likely contributed by its passive antioxidants in the form of enriched flavonoids and selenium content (Ponnusamy et al., 2008). Among the triterpenes isolated from *Centella* plant, asiaticoside is the most abundant and responsible for stimulating the antioxidant activity in the early phase of the wound healing process (Shukla et al., 1999). *In vitro* studies have found high antioxidant activity of leaves of *Centella* in three pathways: superoxide free-radical activity (86.4%), inhibition of linoleic acid peroxidation (98.2%), and 2,2-diphenyl-1-picryl-hydrazyl (DPPH) radical scavenging activity (92.7%; Vimala et al., 2003; Pittella et al., 2009). In addition to its antioxidant activity, asiaticoside has been reported as being helpful in reducing dementia and enhancing cognition (De Souza et al., 1990) and found to have therapeutic value against β-amyloid neurotoxicity (Mook-Jung et al., 1999). Protection from oxidative damage, apart from encompassing both the direct passive antioxidants and the stimulants of enzymes involved in antioxidant defense, also encompasses a reduction in monamine levels because these compounds can spontaneously auto-oxidize to free radicals (Bindoli et al., 1992). *Centella* is useful even in this way of monamine reduction to counter oxidative damage. There is concern that *Centella* might cause liver damage in some people and cause side effects like stomach upset, nausea, and itching (Jorge and Jorge, 2005).

*Asparagus racemosus* Wild (Asparagus; Shatavari, Shatuli, Vrishya) Geographical distribution and ethnomedical description: *A. racemosus* is commonly known as Shatavari in India. Romans used *A. racemosus* for food and medicinal purpose. It was first cultivated in England at the time of Christ and brought to America by the early colonists. Shatavari has great reputation in ayurveda for relief in uterine diseases, to increase lactation, as an antacid, and as a tonic. Chemical constituents: The major bioactive constituents of Asparagus are a group of steroidal saponins. This plant also contains vitamins A, B1, B2, C, E, and folic acid. Asparagus also contains metals like Mg, P, Ca, and Fe. Other primary chemical constituents of Asparagus are essential oils, asparagine, arginine, tyrosine, flavonoids (kaempferol, quercetin, and rutin), resin, and tannin (Bopana and Saxena, 2007). Shatavarins I–IV, major steroidal saponins are present in the roots. Five steroidal saponins, shatavarins VI–X, shatavarin V, immunoside, and schidigerasaponin D5, have been isolated from the roots of *A. racemosus* (Hayes et al., 2008). Other active compounds such as quercetin, rutin, and hyperoside are found in the flowers and fruits, while diosgenin and quercetin-3 glucuronide are present in the leaves (Asmari et al., 2004). *Asparagus* is also rich in phytoestrogens, namely the isoflavones and coumastans (Bopana and Saxena, 2007).

#### Active antioxidant and other roles

Shatavari is used for sexual debility and infertility in both sexes in some modern studies. It has been used to ameliorate menopausal symptoms (Thakur et al., 1989). The herb is widely reported for galactagogue activity (Atal and Kapur, 1982; Thakur et al., 1989; Shah and Qadry, 1990). It is also known for its nootropic effects (Ojha et al., 2010). Further, methanolic Asparagus extract dose-dependently inhibited acetylcholinesterase enzyme in specific brain regions (prefrontal cortex, hippocampus, and hypothalamus; Ojha et al., 2010). These activities may be related to the augmentation of cholinergic transmission as methanolic Asparagus extract significantly inhibited the enzyme cholinesterase. Gindi (2011) further explored these nootropic effects by using scopolamine-induced memory deficit and AlCl_3_ induced cognitive deficits in rats. The nootropic effects could be in part due to the observed increase in the antioxidant activity in terms of DPPH radical and NO radical scavenging activity (Gindi, 2011).

The extracts of *A. racemosus* protect against kainic acid induced hippocampal and striatal neuronal damage that is accompanied by an increase in lipid peroxidation and protein carbonyl content, decline in GPX activity, and reduced glutathione (GSH) content (Parihar and Hemnani, 2004). The *Asparagus* extract supplemented mice show an improvement in the GPX activity and the GSH content and a reduction in the membrane lipid peroxidation and protein carbonyl content (Parihar and Hemnani, 2004). Similar to kanic acid induced insult, immobilization stress induced oxidative changes in the hippocampal subregion are also counteracted by the antioxidant effects of *Asparagus* extracts (Vimal et al., 2010). Treatment with the *Asparagus* extract compensates the immobilization stress induced decrease in the SOD, catalase, ascorbic acid, and lactate dehydrogenase levels and lowers the heightened MDA levels (Vimal et al., 2010). Renal pain, peripheral edema, exacerbation of gout, and skin allergies have been reported for a small number of patients (Chrubasik et al., 2006). Lipid transfer proteins, profilin, and glycoproteins may account for the reactions as well as for cross-sensitivities of *Asparagus* (Pajno et al., 2002; Rieker et al., 2004; Tabar et al., 2004; Volz et al., 2005).

### *Acorus calamus* Linn. (sweet flag, calamus; bach, vacha)

#### Geographical distribution and ethnomedical description

*Acorus calamus* L. is a polyploidic marsh plant indigenous to Asia that is now distributed along trade routes all over the northern hemisphere. The aromatic rhizome has been widely used as an herbal remedy. Ayurvedic medicine and traditional Chinese medicine use the drug to treat CNS related diseases such as epilepsy and insomnia (Williamson, 2002; Khare, 2004). In European folk medicine, *A. calamus* rhizomes have been mainly used to alleviate gastrointestinal ailments such as acute and chronic dyspepsia, gastritis and gastric ulcer, intestinal colic, and anorexia.

#### Chemical constituents

Its extract contains monoterpenes, sesquiterpenes, phenylpropanoids, range of sesquiterpene hydrocarbons, alcohols, and ketones (e.g., acorone, acoragermacrone, calamendiol) besides eugenol, methyl isoeugenol, and phenyl propane derivatives such as α and β-asarone (Raina et al., 2003). Interestingly β-asarone content in *Acorus* is possibly dependent upon the genetic composition of plant as the volatile oil from the tetraploid “Indian” form of calamus contains β-asarone as a major component (up to 95%) whereas the oil from the “European” or triploid form contains less than 10% β-asarone (Lander and Schreier, 1990). A diploid form of the plant contains virtually no β-asarone (Lander and Schreier, 1990).

#### Active antioxidant and other roles

Several *in vivo* studies support a sedative and tranquilizing action of the extracts of *A. calamus* (Dandiya et al., 1959; Dandiya and Menon, 1963; Vohora et al., 1990). The tranquilizing effect has been suspected to be due to α-asarone and β-asarone (Dandiya and Menon, 1963; Liao et al., 1998; Zanoli et al., 1998) but up to now the underlying mechanisms of action of these compounds has not been convincingly demonstrated. The ethanolic extract of *Acorus calamus* has been shown in a report to prevent acrylamide-induced hind limb paralysis, decrease GSH and GST, and increase dopamine receptors in the corpus striatum (Shukla et al., 2002). *Acorus* is effective in attenuating the noise stress via scavenging free radicals and lowering the lipid peroxidation in many regions of the rat brain, like cerebral cortex, cerebellum, pons-medulla, midbrain, hippocampus, and hypothalamus (Manikandan et al., 2005). *Acorus calamus* extract exhibit radioprotection abilities in part due to antioxidant activity by increasing the activities of major enzymes of the antioxidant defense system, especially SOD, CAT, and GPX and the levels of GSH and decrease in the formation of MDA and in part due to the reduction in DNA strand breaks (Sandeep and Nair, 2010).

### *Bacopa monnieri* (Linn) *wettst*. (Coastal waterhyssop; Nirbrahmi)

#### Geographical distribution and ethnomedical description

*Bacopa monnieri* is commonly found in wet marshy and damp places throughout India. Occasionally it is also referred to Brahmi, the same common Hindi name as that of *C. asiatica*. A disambiguating and more appropriate Hindi name is Nirbrahmi (Nir = water + Brahmi) indicating the marshy habitat of *Bacopa*. The ambiguity, likely introduced in the sixteenth century, still persists in the common usage due to a big overlap in the medicinal value of the two herbs. *B. monnieri* is a major constituent of the traditional Medhya rasayana (Medhya = intelligence, Rasayana = rejuvenators) formulations, which are considered to facilitate learning and improve memory (Garai et al., 1996). In traditional medicine, the plant is used as nervine tonic, diuretic, and to treat asthma, epilepsy, insanity, and hoarseness.

#### Chemical constituents

Pharmacological compounds from *B. monnieri* include alkaloids, saponins, and sterols. It also contains the brahmine, herpestine, saponins, monnierin, hersaponin, bacosides, four aglycones of ebelin, lactone (Kulshreshtha and Rastogi, 1973), bacogenins A_1_ (Kulshreshtha and Rastogi, 1973; Chandel et al., 1977), jujubogenin, and pseudojujubogenin (Kawai and Shibata, 1978), d-mannitol, betulic acid, β-sitosterol, stigmasterol and its esters, heptacosane, octacosane, non-acosane, triacontane, hentriacontane, dotriacontane, nicotine, 3-formyl-4-hydroxy-2H-pyran, and luteolin and its derivatives (Rastogi and Mehrotra, 1990).

#### Active antioxidant and other roles

*Bacopa monnieri* extract shows neuroprotective effect against neurotoxicants like aluminum and nicotine in experimental animals (Jyoti and Sharma, 2006; Vijayan and Helen, 2007). In one study, *B. monnieri* extract was shown to reduce the amyloid levels in a Alzheimer’s disease model mice, suggestive of its therapeutic potential (Holcomb et al., 2006) and we think further studies are needed to explore this issue further. In one study, the oral administration of 5–10 mg *B. monnieri* crude extract per kg body weight markedly reduced the memory deficits in rats along with a reduction in acetylcholine concentrations, choline acetylase activity, and muscuranic receptor binding in the hippocampus and the frontal cortex (Dhawan and Singh, 1996). *B. monnieri* also helps in coping with combined hypoxic, hypothermic, and ischemic stress that could lead to the onslaught of free radicals (Rohini et al., 2004). Studies suggest that *Bacopa* extracts scavenge superoxide anion and hydroxyl radical and reduce H_2_O_2_ induced cytotoxicity and DNA damage in human fibroblast cells (Tripathi et al., 1996; Rai et al., 2003). *B. monnieri* extracts modulate the expression of certain enzymes involved in the generation and the scavenging of ROS in the brain (Govindarajan et al., 2005). Bacosides from *B. monnieri* are known for their antioxidant activity (Tripathi et al., 1996; Bhattacharya et al., 2000; Deb et al., 2008), for example bacoside A has been shown to offer significant protection against chronic cigaret smoking-induced oxidative damage in rat brains (Anbarasi et al., 2006). The antioxidant activity of *B. monnieri* is able to explain, at least in part, the reported antistress, cognition-facilitating, and ant-aging effects produced by it in experimental animals and in clinical situations. In a study, when a preparation of the plant was evaluated for safety and tolerability it showed no serious adverse effects but there were some reports of mild gastrointestinal symptoms (Pravina et al., 2007).

### *Celastrus peniculatus* Willd. (climbing staff tree, intellect tree; Mal-kangani)

#### Geographical distribution and ethnomedical description

*Celastrus paniculatus* is a large, woody, climbing shrub, distributed almost all over India. In traditional medicine the seeds are considered to be stimulant, intellect promoting, laxative, emetic, expectorant, appetizer, aphrodisiac, cardiotonic, anti-inflammatory, and diuretic (Chopra and Chopra, 1958; Warrier et al., 1996; Bhanumathy et al., 2010). It has also been traditionally used for healing abdominal disorders, leprosy, pruritus, skin diseases, paralysis, cephalalgia, arthralgia, asthma, leucoderma, cardiac debility, inflammation, nephropathy, amenorrhoea, dysmenorrhoea, beri-beri, and sores (Chopra and Chopra, 1958; Warrier et al., 1996; Bhanumathy et al., 2010). In traditional medicine the bark is considered to be an abortifacient, a depurative and a brain tonic and the leaves as emmenagogue and the leaf sap, a good antidote for opium poisoning (Chopra and Chopra, 1958; Warrier et al., 1996; Bhanumathy et al., 2010).

#### Chemical constituents

*Celastrus peniculatus* contains several possible active ingredients like polyalcohols, malkanguniol (Hertog et al., 1974), β-sitosterol, β-amyrin, a pentacyclic triterpene diol paniculatadiol (Hertog et al., 1973), several phytosteroids, saponins, and tannins (Joshi and Sabnis, 1989), *n*-triacontanol, pristimerin (Jain, 1963), celastrol, pristimerin, zeylasterone, and zeylasteral (Gamlath et al., 1990).

#### Active antioxidant and other roles

*Celastrus peniculatus* has been reported for stimulatory effects on brain (Warrier et al., 1996). In an old study it was found to enhance memory and improve the IQ of mentally retarded children (Morris et al., 1953). The seed extract of *C. paniculatus* demonstrate improvements in learning abilities in mice along with improvements in antioxidant activity in the form of significant decrease in the brain levels of MDA and increases in levels of glutathione and catalase (Kumar and Gupta, 2002). *Celastrus* extracts can attenuate hydrogen peroxide and glutamate-induced injury in embryonic rat forebrain neuronal cells, in a dose dependent manner (Godkar et al., 2006). SOD activity was unaffected after treatment, in comparison to this catalase activity was increased and MDA levels were reduced (Godkar et al., 2006).

### *Convulvulus pleuricaulis* Choisy. (Bindweed; Kaudiali, Shankhahuli, Shankhpushpi)

#### Geographical distribution and ethnomedical description

*Convolvulus pleuricaulis* is used in traditional systems of medicine in anxiety, neurosis, insanity, epilepsy, insomnia, fatigue, low energy level, and as a brain tonic (Sethiya and Mishra, 2010). Traditionally the whole herb is used medicinally in the form of decoction with cumin and milk in nervous debility, and loss of memory (Chopra and Chopra, 1958; Warrier et al., 1996). This plant is widely distributed across Indian subcontinent.

#### Chemical constituents

*Convolvulus* contains neuroactive alkaloids such as shankhpushpine, convolamine, and other likely potent compounds such as hextriacontane (Deshpande and Srivastava, 1975), scopoletin, *P*-sitosterol and ceryl alcohol (Deshpande and Srivastava, 1969), 20-oxodotriacontanol, tetratriacontanoic acid and 29-oxodotriacontanol, flavonoid kampferol and phytosteroids such as phytosterol and *P*-sitosterol (Singh and Bhandari, 2000).

#### Active antioxidant and other roles

Supporting the long-standing belief and extensive large-scale commercial use in India of the aqueous extract of *Convolvulus*, a recent rodent study showed significant improvement in learning by finding reduction in scopolamine induced increase in the transfer latency in the elevated plus maze and improvement in the impaired spatial memory in the Morris water maze (Bihaqi et al., 2011). Extract was also found to decrease acetylcholinesterase activity in the cortex and hippocampal subregion and increase the activity of glutathione reductase, SOD, and GSH (Bihaqi et al., 2011). Whether the learning boosting effects were solely due to antioxidant activity in the above-mentioned study is not obvious but the potential of this plant for finding active stimulants of antioxidant pathway seems very high. Significant antioxidant boosting and acetylcholinesterase inhibitory properties have previously been observed too (Nag and De, 2008). Extracts of convolvulus have also been shown to reduce the increased levels of MDA along with altering glutathione levels in hippocampus (Parihar and Hemnani, 2003). In summary, studies demonstrate the potential of *C. pleuricaulis* for being a promising source of novel stimulants of antioxidant responses.

### *Curcuma longa* Linn. (Turmeric; Haldi)

#### Geographical distribution and ethnomedical description

*Curcuma longa* known commonly as Haldi is perennial rhizome plant grows also all over India. Its antioxidant potential is well demonstrated and many compounds commercialized. Given the ease of availability of information on this plant (Ammon and Wahl, 1991; Park and Kim, 2002; Singh et al., 2002; Basnet and Skalko-Basnet, 2011; Schaffer et al., 2011; Mythri and Bharath, 2012), we are keeping discussion of this herbal source to a minimum.

#### Chemical constituents

*Curcuma* contains phenylheptanoids, monoterpenes, ses-quiterpenes, several diphenyl alkanoids, phenyl propene derivatives of cinnamic acid type, and terpenoids, along with very active and well studied compound: curcumin (Roth et al., 1998; Park and Kim, 2002; Singh et al., 2002). The rhizomes contains curcuminoids, curcumin, demethoxy curcumin, bis-demethoxycurcumin, 5′-methoxycurcumin, and dihydrocurcumin that are known antioxidants (Ravindranath and Satyanarayana, 1980; Kiuchi et al., 1993; Nakayama et al., 1993; Park and Kim, 2002).

#### Active antioxidant and other roles

Methanol extract of turmeric has led to the isolation of Calebin-A and some curcumin compounds that are claimed to offer a good degree of protection to the neuronal cells against β-amyloid deposition (Park and Kim, 2002). Oral administration of curcumin to alcohol-fed rats causes a significant reversal of brain lipid peroxidation, indicating its neuroprotective role (Rajakrishnan et al., 1999). Curcuminoids have been found to be potent inhibitors of lipid peroxidation, inhibiting lipid peroxidation in rat brain homogenates, and rat liver microsomes to a higher level than alpha-tocopherol (Rao, 1994). It is not clear whether the antioxidant potential of turmeric is due to rich passive antioxidants that it contains or due to additional active antioxidant stimulating compounds. While the use of turmeric in food and monitored quantities as medicine is very safe, very high doses of *C. longa* (500 mg/kg) have been reported to induce chromosomal aberrations in animal models (Jain et al., 1987), suggesting need for caution when selecting the medicinal dose.

## Bioavailability of Herbal Drugs and Antioxidant Property

Bioavailability has been broadly defined as “absorption and utilization of a nutrient” (Krebs, 2001). The degree and quantity of penetration of herbal drug or its active ingredient is determined by its bioavailability (Youdim et al., 2004; Reddy et al., 2005). Bioavailability of ayurvedic herbal drugs has been largely neglected and only some studies have conducted fine quantitative measurements, for example measurement of baicalein found in *T. arjuna* (Tilak et al., 2006). Bioavailability can depend on chemical complexity of the herb due to synergistic and antagonistic actions of the constituents in promoting absorption, hydrophobic properties determining the ability to cross luminal wall, gut microflora, and hepatic activity of the individual, and chemical modifications of the herbal constituents. Constituents in herbal drugs must cross the blood brain barrier to be effective in the CNS and there is a dearth of literature on Indian herbs, especially ones with bodily antioxidant stimulating potential on such an important topic. Synergistic interactions of herbs can play an important role in bioavailability, like capsicum can increase the availability of theophylline (Bouraoui et al., 1986) and tamarind can increase the bioavailability of Asprin (Mustapha et al., 1996). Atal et al. (1981) demonstrated that long pepper, black pepper, and ginger can increase bioavailability of some compounds. There is evidence of considerable person-to-person variation in gut microflora and hepatic activity too (Williamson and Manach, 2005). A look at turmeric extracts in the context of non-oxidative issues can bring to light the problem of a compound or an extract working *in vitro* failing to work *in vivo* because of issues of bioavailability (Basnet and Skalko-Basnet, 2011; Belkacemi et al., 2011; Irving et al., 2011).

## Conclusion

While ayurvedic plants have been explored for many passive antioxidants and medical uses for serious neurological diseases such as Alzheimer’s disease (Anekonda and Reddy, 2005), a systematic analysis for the herbs covered in this review for the ability to increase body’s ability to reduce antioxidant stress has been previously missing. In the present review we provide bodily antioxidant stimulating potential of selected Indian medicinal plants used in traditional medicine system of ayurveda. We speculate that the cases where traditional medicine actually works successfully beyond a placebo effect, it is likely due to the effect of herbal extracts on multiple nodes of the signaling pathway, instead of single target approach of the Western medicine. Our focus has been on plants that can likely yield direct stimulants of physiological antioxidant response. Reasons to identify, synthesize, and modify the active compounds of traditional herbs instead of using the traditional formulae alone are as follows:

1. Many of the supposed effects of some traditional medicine are simply placebo and it is important to disambiguate real effects of herbs from placebo effects.
2. In absence of concentrating the active compounds beyond extracts most traditional medicines usually can only aid slow recovery from disease but do not offer fast solutions.
3. The extracts of a plant can have components that have opposing effects. For example *C. asiatica* is rich in many pro-oxidative metals, while being a rich source of passive and active antioxidants.
4. Many of the components of certain plants can be allergens to some patients and isolation of active components would eliminate allergens and other contaminants.
5. Many of the passive antioxidants that are known to be good antioxidants *in vitro* are considered to be at best just weak antioxidants *in vivo*. In fact, some studies suggest that pro-oxidative activities of flavanoids outweigh antioxidant activities *in vivo* (Procházková et al., 2011). This necessitates the need for finding agents that can stimulate body’s own antioxidant response. Most of the plants that we cover have been shown to stimulate intrinsic antioxidant responses in humans.

While it is important to isolate and modify the active compounds from Indian pharmacopeia, it is equally important to understand that some of the efficacy of these preparations may be due to multiple active components that act synergistically in herbal concoctions, hence a single molecule stimulant of antioxidant responses may not be the end point in pharmaceutical development for all the above-mentioned plants.

While we have focused on traditional medicines that have been clinically tested and evaluated by modern means, we would like to point out that the technical finesse and rigor of many studies leaves a large room of improvement. Funding issues and cultural biases mean that currently most cutting edge laboratories shy away from exploring these traditional medicines. Many of the clinical studies that have looked at the Indian traditional medicine, have so far been small scale and many have lacked a systematic evaluation of multiple parameters of patient health that are increasingly becoming the norm for clinical trials. Clinical studies using traditional medicine have also rarely been double blind leaving chance of physician’s bias in some of the reported studies. While the state-of-the-art exploration of these herbal sources has been a minority of research in this field so far, we expect significant improvement in research the near future. The reason to expect such a change, apart from cultural and attitude shift amongst biologists is the dire need of active antioxidants that can help patients deal with many chronic ailments, aging problems, and neurodegenerative disorders. While Western medicine has failed on most counts to deliver such active antioxidants, modern studies of many ayurvedic plants mentioned in this review have been very promising, necessitating their future exploration. We expect that a modern systems biology approach toward studying physiology, epigenomics, transcriptomics, proteomics, and metabolomics, apart from standard reductionist approaches, will be employed in exploring the potential of few traditional medicines that we have enumerated in this review.

## Conflict of Interest Statement

The authors declare that the research was conducted in the absence of any commercial or financial relationships that could be construed as a potential conflict of interest.
